# BPA and Insulin Resistance: Evidence of Effects in Dams and Offspring

**DOI:** 10.1289/ehp.118-a399a

**Published:** 2010-09

**Authors:** M. Nathaniel Mead

**Affiliations:** **M. Nathaniel Mead**, a science writer living in Durham, NC, has written for *EHP* since 2002

Recent population studies have associated increased exposure to persistent organic pollutants with an elevated risk of insulin resistance and thus a greater likelihood of developing type 2 diabetes and heart disease. Among these chemicals is bisphenol A (BPA), a pervasive endocrine-disrupting compound used to make polycarbonate plastic and epoxy resins. Now researchers report evidence that exposure to environmentally relevant doses of BPA during pregnancy may alter insulin sensitivity and glucose homeostasis in mice, with potential disease-related consequences for both the mother and her male offspring **[*EHP*** **118(9):1243–1250; Alonso-Magdalena et al.]**.

The study evaluated the effects of two different doses of BPA (10 or 100 μg/kg/d) administered to pregnant mice during days 9–16 of gestation. Glucose metabolism experiments were performed on the mice during pregnancy and subsequently on their offspring.

BPA exposure aggravated the insulin resistance that occurs during pregnancy, and four months postpartum, BPA-treated mice weighed more and had more severe insulin resistance than untreated females. The BPA-treated mice also showed elevated plasma levels of insulin, leptin, triglycerides, and glycerol (a breakdown product of triglycerides), as well as molecular changes indicating reduced insulin sensitivity in skeletal muscle and liver.

Given that levels of the hormone leptin are normally increased during pregnancy, the authors propose that future research should seek to determine whether BPA directly regulates leptin release from fatty tissue or whether the observed hyperleptinemia is a consequence of the altered metabolic state of these animals.

Previously the same research team had shown a relationship between BPA exposure and glucose intolerance and insulin resistance in adult male mice. In the present study, they further observed that, at 6 months of age, male offspring exposed to BPA *in utero* had reduced glucose tolerance, increased insulin resistance, and altered blood parameters compared with offspring of untreated mothers. Moreover, studies of the male offspring’s pancreases showed altered calcium signaling and insulin secretion.

The authors conclude that BPA exposure during pregnancy can alter the mother’s glucose metabolism during pregnancy and later in life, and may contribute to metabolic disorders relevant to glucose homeostasis in the male offspring. The findings also suggest that BPA exposure should be further examined as a risk factor for diabetes.

